# Locus Characterization and Gene Expression of Bovine FNDC5: Is the Myokine Irisin Relevant in Cattle?

**DOI:** 10.1371/journal.pone.0088060

**Published:** 2014-01-31

**Authors:** Katrin Komolka, Elke Albrecht, Lisa Schering, Julia Brenmoehl, Andreas Hoeflich, Steffen Maak

**Affiliations:** 1 Institute for Muscle Biology and Growth, Leibniz Institute for Farm Animal Biology (FBN) Dummerstorf, Dummerstorf, Germany; 2 Institute for Genome Biology, Leibniz Institute for Farm Animal Biology (FBN) Dummerstorf, Dummerstorf, Germany; Faculty of Animal Sciences and Food Engineering, University of São Paulo, Pirassununga, SP, Brazil, Brazil

## Abstract

The transmembrane protein FNDC5 was recently characterized as precursor of an exercise induced myokine named irisin. Previous studies found a relationship between circulating irisin levels and muscle mass in humans. Consequently, we tested the hypothesis whether FNDC5/irisin is involved in the modulation of body composition in cattle. Since information on the bovine FNDC5 locus was scarce, we characterized the gene experimentally as prerequisite for these investigations. We provide here a revised and extended gene model for bovine FNDC5. Although similarly organized like the human and murine loci, a higher variability was observed at transcript level in the bovine locus. FNDC5 mRNA was abundant in bovine skeletal muscle and was detected at lower levels in adipose tissue and liver. There were no expression differences between two groups of bulls highly different in muscularity and adiposity. Full-length FNDC5 protein (25 kDa) was present in bovine skeletal muscle independent of muscularity. Neither FNDC5 nor its putatively secreted peptide irisin were found in circulation of bulls. In contrast, we demonstrated that FNDC5 (25 kDa) and irisin (12 kDa) were present in murine skeletal muscle and that irisin was circulating in murine serum. This indicates fundamental differences in the regulation of FNDC5 and irisin between rodents and cattle.

## Introduction

Fibronectin type III domain containing 5 (Fndc5) was originally described as crucial factor for cellular differentiation of skeletal muscle in embryonic mice. It was predominantly detected in peroxisomes [1]. In contrast, strong expression in murine, embryonic brain but a weak signal in murine skeletal muscle was observed in another study [2]. It was not until a decade later that Fndc5 attracted attention again, when Boström et al. [3] characterized it as precursor of a protein named irisin. Mice with transgenically elevated expression of peroxisome proliferator-activated receptor gamma, coactivator 1 alpha (Ppargc1a) in skeletal muscle responded with a dramatically increased Fndc5 mRNA expression. As a result, a brown fat-like expression signature was observed in white, subcutaneous adipocytes. A weaker but similar effect was observed in wild-type mice after 3 weeks of free wheel running. The authors hypothesized that Fndc5 protein was synthesized as type-I membrane protein which was cleaved proteolytically. The resulting N-terminal fragment - called irisin - was assumed to be released into circulation. Irisin was increased after physical activity and suggested to mediate - at least in part - beneficial effects of exercise on metabolism [3].

Shan et al. [4] demonstrated that myostatin (Mstn) knock-out in mice promoted properties of brown/beige adipocytes in white adipose tissue. This process was non-cell autonomous but caused by Fndc5/irisin secreted from skeletal muscle. The authors provided evidence for a direct activation of the AMPK-Ppargc1a-Fndc5 pathway by suppression of Mstn. First investigations in humans suggested muscle mass as main predictor of circulating irisin [5], [6]. These findings predestined FNDC5 and its putative cleavage product irisin for investigations in meat producing farm animals especially in cattle where genetic variants in MSTN strongly influence body composition and meat quality [7]–[10].

However, subsequent studies on relationships between mRNA and protein abundance of FNDC5 on the one hand and physiological parameters in humans on the other hand revealed contradictory results. Liu et al. [11] confirmed the positive relationship between body mass index and circulating irisin in healthy individuals whereas diabetic patients had significantly lower irisin levels as described in previous studies [5], [6], [12]. In contrast, Timmons et al. [13] could not detect an induction of FNDC5 mRNA by physical exercise in humans. This was confirmed by Pekkala et al. [14] who concluded that observed variations in FNDC5 abundance were likely random. A recent study revealed that human full-length FNDC5 could be translated from an alternative AUA initiation site only [15]. Since full-length expression of FNDC5 is prerequisite for irisin synthesis, Raschke et al. [16] investigated the translation efficiency of the transcript derived from this alternative codon and found a reduction to 1% compared to a construct containing a regular start codon. They concluded that irisin cannot be produced in humans in noteworthy amounts. Interestingly, a regular start codon is present in all other species [15] thus allowing production and secretion of irisin. However, in none of the studies in any species the peptide irisin was detected at its expected size of 12.6 kDa up to date as was noted by several authors [16]–[19]. Instead protein bands of 20–26 kDa, likely corresponding to full-length FNDC, were shown in mice, rats, and humans and were differently termed as irisin, FNDC5 or soluble FNDC5 [3], [4], [17], [20], [21]. Against the background of inconsistent data on FNDC5/irisin, we analyzed FNDC5/irisin expression in muscle and plasma of cattle with highly different muscularity and intramuscular fat deposition in this study. Since the predicted bovine FNDC5 gene deviates strongly in terms of exon structure from that in rodent and human genomes according to the current bovine genome annotation, we analyzed the structure of the bovine FNDC5 locus experimentally as prerequisite for further investigations.

## Materials and Methods

### Animals

Two groups of bulls (total n = 20) were selected from a F_2_-population (Charolais × German Holstein) comprising 248 bulls [22]. The animals were selected for either high or low muscularity and fatness traits (protein content of the carcass; intramuscular fat content in longissimus muscle; Table 1) from both opposite ends of the distribution of the traits in the population, respectively. Carcass composition, longissimus muscle weight and intramuscular fat content were determined as described by Pfuhl et al. [23]. Animals were tethered on individual feeding places with semi-slatted floor and automatic drinking bowls under approved conditions (State Office for Agriculture, Food Safety and Fishery; Permit number: LALLF M-V/TSD/7221.3-2.1-010/03) and slaughtered in the slaughterhouse of the Leibniz Institute for Farm Animal Biology (FBN) Dummerstorf, Germany (EEC Approval Number ES1635/EZ1635) at an age of 18 months. Samples were collected from M. longissimus dorsi (LM), M. semitendinosus (SM), subcutaneous adipose tissue (SAT) and liver immediately after slaughter and stored in liquid nitrogen until further processing.

**Table 1:** Body composition of bulls with high and low muscularity in comparison with the total population.

Group	High muscularity	Low muscularity	Total population
**Trait/Number (n)**	10	10	248
Live weight at slaughter, kg	702±30^a^	688±46^a^	690±61
Weight of longissimus muscle (LM), kg	8.4±0.8^a^	7.3±0.6^b^	8.1±0.9
Intramuscular fat content, % in LM	1.7±0.4^A^	6.8±2.4^B^	3.4±1.7
Calculated protein content of carcass, %	15.2±0.7^A^	13.3±0.6^B^	14.5±0.8
Calculated fat content of carcass, %	12.8±2.7^A^	21.7±3.5^B^	16.4±3.9

Different superscripts indicate significant differences between high and low muscularity groups (a, b: p<0.05; A, B: p<0.01).

### RNA and Protein Isolation

Total RNA was isolated from LM and liver using QIAzol lysis reagent and chlorophorm isopropanol precipitation as described by the manufacturer (Qiagen, Hilden, Germany). For LM samples, we added a purification step with the NucleoSpin Extract II reagent (Macherey-Nagel, Dueren, Germany) according to manufacturer’s guidelines. RNA from SAT samples was isolated using the RNeasy Lipid Tissue Mini Kit (Qiagen) as described by the manufacturer. Total protein of LM was extracted using CelLytic MT lysis reagent (Sigma-Aldrich, Munich, Germany) and a protease inhibitor according to manufacturer’s protocol. RNA and protein concentrations were quantified with a NanoDrop ND-1000 spectrophotometer (Peqlab, Erlangen, Germany). Additionally, RNA integrity was determined with an Experion Automated Electrophoresis System using the RNA StdSens analysis chip (Bio-Rad, Munich, Germany). RNA quality indices (RQI) values of >7 indicated sufficient RNA quality.

Samples of serum and protein from M. quadriceps femoris from a single wild type mouse were available as positive control in western blots.

### cDNA-PCR

Synthesis of cDNA was performed with 100 ng total RNA of LM, SAT and liver, respectively using the iScript cDNA Synthesis Kit (Bio-Rad) with 20 µl reaction volume according to manufacturer’s instructions. We performed qualitative detection of FNDC5 transcripts in LM in a cDNA-PCR approach. Transcript specific primers (Table 2) were designed with Primer 3 software (v. 0.4.0., http://frodo.wi.mit.edu/primer3/) and synthesized by Sigma-Aldrich. Target sequences were amplified in a 10 µl reaction volume containing 10 ng cDNA, 2 mM of the respective primer pair, and 1× PCR master mix (Thermo Scientific, Vilnius, Lithuania) in a pecSTAR 96 Universal thermocycler (Peqlab) with standard PCR protocol as described elsewhere [24]. The amplicons were subjected to electrophoresis on 3.0% agarose gels containing ethidium bromide and were visualized under ultra violet light.

**Table 2:** Primer sequences for cDNA amplification and transcript sequencing.

Transcript	Sequence (5′-3′) forward	Amplifiedexons^1^	AmpliconLength [bp]	T_A_ (°C)
	Sequence (5′-3′) reverse			
T1–T1	TGGCTGGGCTGCGTCT	2-3a(b)-4-5-6b-6d	605 (601)	60
	GAGCAAGCACTGAAAGGGTT			
T1–T2	TGGCTGGGCTGCGTCT	2-3a(b)-4-5-6b-6e(long)	666 (662)	60
	CAGGGACCAGCAGAGAAGAA			
5′-T2	TGGCTGGGCTGCGTCT	2-3a(b)-4	282 (278)	60
	CTGACCCTGGATGGATATGG			
5′-T3	TGGCTGGGCTGCGTCT	2-3c-4-5	487	62
	CACATGAACAGGACCACGAC			
3′-T3	ATCATCGTCGTGGTCCTGTT	5-6b-6e(short)	873	58
	TGTCCATGATGAATGGCTTG			
3′-T4	GGTAAGCTGGGATGTCTTGG	3-4-5-6c	772	60
	GTCACACAGCAGAGCAGGAG			

1 :Exon numbers according to Figure 2B.

T_A_: annealing temperature.

### RT-qPCR

Quantitative gene expression measurements were done in duplicate in a volume of 10 µl per reaction containing 10 ng cDNA, 2 mM of the respective forward and reverse primers (Table 3) and 5 µl SYBR Green Supermix (Bio-Rad) in the iCycler MyiQ 2 with iQ detection system (Bio-Rad). The qPCR basis protocol included an initial denaturation step (95°C for 3 min) followed by 45 cycles (95°C for 10 s, 60°C for 30 s, 70°C for 45 s) and a final melting curve analysis. Crossing point (C_P_) values were determined automatically by iQ5 Software (Version 2.1.97.1001, Bio-Rad). The amplification efficiency E was calculated as E = 10^−1/slope^ of standard curve derived from six serial dilutions (1∶1, 1∶10, 1∶50, 1∶100, 1∶500, 1∶1,000). The expression values were normalized to beta-2-microglobulin (B2M), glyceraldehyde-3-phosphate dehydrogenase (GAPDH) and topoisomerase II beta (TOP2B) using efficiency-corrected ΔΔ-C_P_ method. Gene expression in the two cattle groups (high vs. low muscularity) were comparatively analyzed using the REST algorithm ([25]; REST 2009, Version 2.0.13, Qiagen).

**Table 3:** Primer sequences for RT-qPCR.

Transcript/Gene	Sequence (5′-3′) forward	GenBank acc. no.	Amplicon Length [bp]
	Sequence (5′-3′) reverse		
FNDC5	GGTAAGCTGGGATGTCTTGG	NM_001105421	187
(all transcripts)	CTGACCCTGGATGGATATGG		
FNDC5	CTGGTGCAGGCGGACAG	this study	211
(5′ - transcript 3a)	CCTCCTCCAGGTCCCAGA		
B2M	CAGCTGCTGCAAGGATGG	NM_173893	184
	ATTTCAATCTGGGGTGGATG		
GAPDH	CAGGTTGTCTCCTGCGACTT	NM_001034034.1	183
	GGTCCAGGGACCTTACTCCT		
TOP2B	AAGAAAACAGCACCGAAAGG	XM_002698750	174
	GAGGTCTGAGGGGAAGAGGT		

### Sequencing

The identity of all amplicons from cDNA-PCR and RT-qPCRs was confirmed by sequencing (ABI PRISM 310 Genetic Analyzer; Life Technologies, Darmstadt, Germany). Sequencing of both strands was performed with the primer pairs used for amplification of the respective fragments. CLC Main Workbench 6.8.4 (CLC bio, Aarhus, Denmark) was used for sequence analysis and alignments.

### Western Blot

The following antibodies were used for FNDC5 and irisin detection: (I) FNDC5-c-term, directed against amino acids (aa) 149–178 of the human protein (AP8746b, BioCat, Heidelberg, Germany); (II) Irisin, directed against full length irisin (aa 32–143; A00170-01-100, Biotrend, Cologne, Germany); (III) FNDC5-Sigma directed against aa 97–151 of the human protein (HPA051290, Sigma-Aldrich).

Twenty micrograms of either muscle protein or plasma protein after albumin removal (Aurum Affi-Gel Blue mini columns, Bio-Rad) were diluted in Laemmli buffer with ß-mercaptoethanol, denatured at 95°C for 5 min, and separated on Criterion TGX 12% gels (Bio-Rad). For determination of the molecular weight of the protein bands, a molecular weight marker (Page Ruler, Thermo Scientific) was separated simultaneously. Proteins were transferred to a polyvinylidene difluoride (PVDF) membrane (Trans-Blot Turbo transfer pack, Bio-Rad) using a semi dry blotter (Trans-Blot, Bio-Rad). For tests of specificity of antibody binding, 15% SDS-PAGE 10×10 cm mini gels were used and blotted on PVDF membranes (Carl Roth, Karlsruhe, Germany). Equal loading of the gels and proper transfer of the proteins to the membranes were verified by Ponceau red staining according to standard procedures. Membranes were blocked for 1 h in either 5% non-fat dry milk in Tris-buffered saline (TBS), for detection of Fndc5, or in 1× Roti-Block (Carl Roth), for detection of irisin. Membranes were incubated with primary antibodies (FNDC5-antibodies diluted 1∶1,000, irisin-antibody diluted 1∶3,000) over night at 4°C. The specificity of antibody binding was tested in parallel blots which were incubated with either the antibody or with the antibody blocked with the respective blocking peptide prior to incubation (FNDC5-c-term blocking peptide, AP8746b, BioCat; recombinant irisin, 00170-01-100, Biotrend). After washing, membranes were incubated with Horseradish peroxidase-conjugated secondary antibody (rabbit IgG TrueBlot, 1∶25,000; 18-8,816, eBioscience, Frankfurt, Germany). Antibody label was detected with chemiluminescence substrate (Immun-Star WesternC, Bio-Rad) and a Chemocam HR-16 imager (INTAS, Göttingen, Germany). LabImage 1D software (Kapelan Bio-Imaging, Leipzig, Germany) was used to quantify intensity of specific bands. Quantification of alpha tubulin (T6074, Sigma-Aldrich, 1∶5,000, secondary antibody rabbit anti-mouse IgG, Calbiochem, Merck, Darmstadt, Germany, 1∶10,000) was used for normalization. After exposure, the antibody was stripped from the membrane with stripping solution (Abcam, Cambridge, U.K.) for 20 min at room temperature to reuse the blot for further antibody incubations.

### Immuno-histochemical Analysis

Muscle (SM) was cryo-sectioned (8 µm thick) using a Leica CM3050 S cryostat microtome (Leica, Bensheim, Germany). Sections were air dried and fixed with 4% paraformaldehyde. After washing with phosphate buffered saline (PBS) and permeabilization with PBS-TritonX100 (PBST), unspecific binding of the secondary antibody was blocked using 10% goat serum in PBST for 15 min. Sections were incubated with the primary antibody against FNDC5-c-term (1∶100) for 1 h at room temperature in a humidity chamber. Specific binding of primary antibody was detected with the respective goat anti rabbit IgG secondary antibody labelled with MFP 488 (MoBiTec, Goettingen, Germany). Nuclei were counterstained with 1 µg/ml Hoechst 33258 (Sigma-Aldrich). Slides were covered using Mowiol mounting medium including 1,4-diazabicyclo[2.2.2]octan (DABCO, Carl Roth) and appropriate cover-slips. Negative controls were incubated either omitting the primary antibody or blocking the primary antibody with the respective peptide. No unspecific binding of the secondary antibody and only minimal unspecific binding of the primary antibody was detected. Immunofluorescence was visualized with a Nikon Microphot SA fluorescence microscope (Nikon, Duesseldorf, Germany) and an image analysis system equipped with CELL^∧^F software and a CC-12 high resolution color camera (OSIS, Muenster, Germany).

### Statistical Analysis

Statistical analysis was performed using the SAS statistical software (Version 9.3, SAS Inst. Inc., Cary, USA). Data were analyzed by ANOVA with fixed factor group (high or low muscularity). The t-test was used for comparisons between groups with *p* ≤ 0.05 as threshold for significant differences.

## Results

### A Revised Gene Model for the Bovine FNDC5 Locus

**Figure 1:**
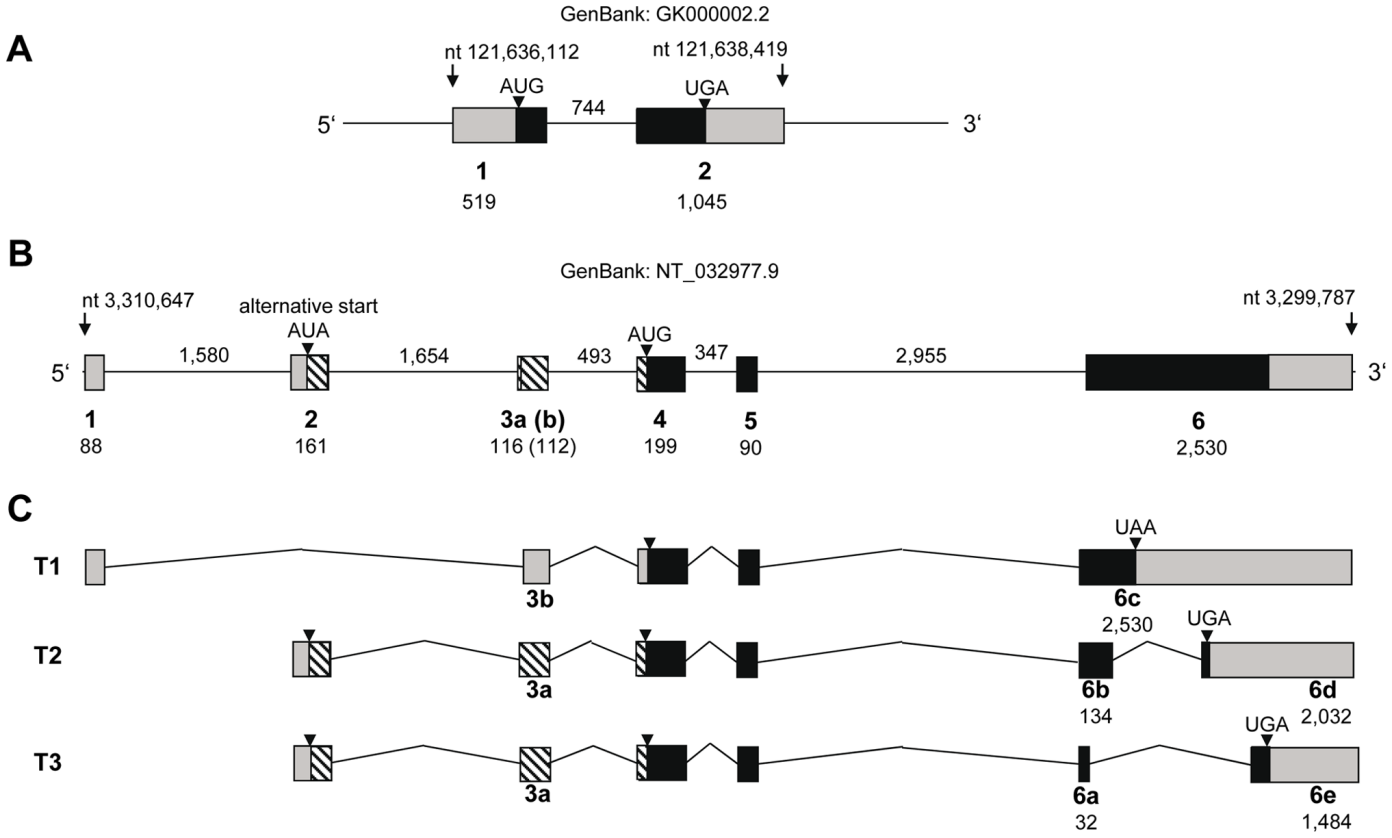
Annotation of the bovine and human FNDC5 genes. (**A**) Bovine FNDC5 locus as annotated in the current build of the bovine genome (UMD 3.1). (**B**) Structure of the human FNDC5 locus. (**C**) Annotated transcripts of the human FNDC5 gene (GenBank accession numbers NM_001171941.1, NM_153756.2, NM_001171940.1). Boxes denote exons with putative coding regions in black. Exon numbers are given below the boxes with lengths in base pairs. Intron lengths are given above the line. Dashed boxes indicate parts of exons which may encode amino acids when the alternative start codon in the human gene is used. The nucleotide numbers of the genomic sequences are given above the arrows.

The bovine gene for FNDC5 was automatically annotated on chromosome (BTA) 2 (GenBank accession number GK000002.2) and comprises two exons. Only the 5′-region of this transcript is supported by a single bovine expressed sequence tag (EST; GenBank accession number EE347697.1) and the exon structure deviates largely from that of the human gene (Figure 1A, B). The human gene consists of 6 exons. Different combinations of 5′- and 3′-exons result in three annotated transcripts, which are supported by human ESTs (Figure 1C).

**Figure 2:**
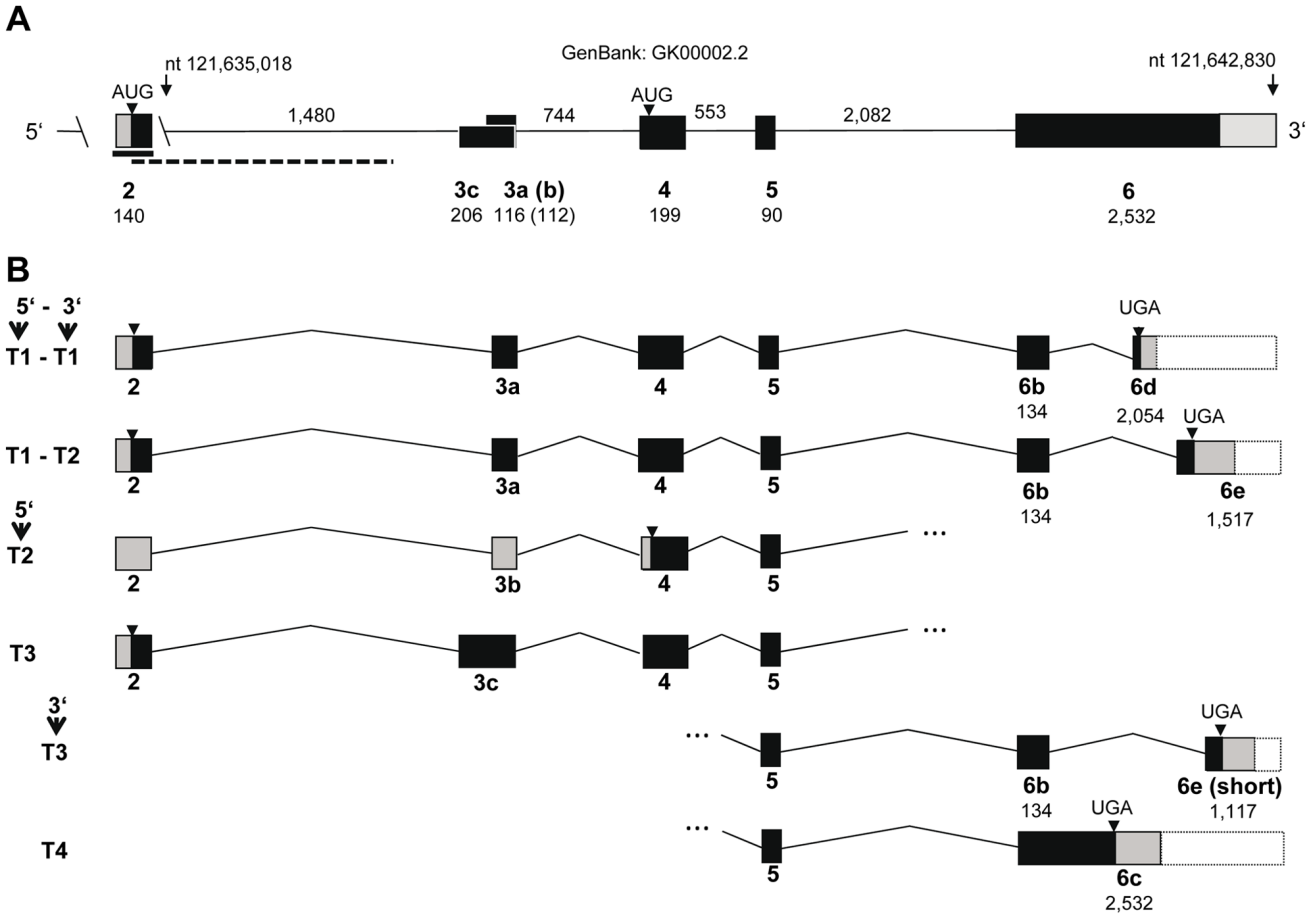
Gene model for bovine FNDC5. (**A**) Genomic organization of bovine FNDC5 on BTA2. Exon 2 (from GenBank accession number EV695596.1, bold line) was anchored to the bovine genome with an overlapping genomic sequence (GenBank accession number AC219791.1, dashed line) and was placed into a gap of the bovine genome. A hitherto unknown exon was named 3c. (**B**) Transcripts identified in bovine skeletal muscle in this study. Boxes denote exons with putative coding regions in black. Grey boxes indicate the proportion of 5′- and 3′-exons which were sequenced in this study. Exon numbers are used like for the human gene and are given below the boxes with lengths in base pairs. Intron lengths are given above the line. Two putative start codons are indicated.

To resolve the obvious conflict between the bovine and human annotation of the FNDC5 locus, we employed a combination of *in silico*-analyses with cDNA amplification and sequencing. We first compared the respective regions on bovine chromosome 2 and human chromosome 1 and identified bovine ESTs mapping to the locus. As expected, the results indicated a similar structure of the bovine and human loci for FNDC5. However, there was no bovine EST with similarity to human exon 1. An exon corresponding to human exon 2 could initially not be aligned because of a gap in the bovine genome sequence. We identified a bovine EST (GenBank accession number EV695596.1) with similarity to human exon 2 and we could align it to the bovine genome by anchoring it to a genomic sequence (GenBank accession number AC219791.1) overlapping with this EST and the bovine genome sequence (Figure 2A, dashed line).

We then identified different transcript variants at 5′- and 3′-ends of bovine FNDC5 in pooled cDNA from skeletal muscle by PCR and subsequent sequencing of the amplicons (Figure 2B). All attempts to amplify the putative exon 1 with any of the downstream exons failed. The inter-species conservation of this region is rather low (75–80% between human, cattle and mouse) and makes the existence of non-coding exon 1 in cattle unlikely. Nevertheless, we retained the same exon numbering in cattle like in humans to avoid confusion.

In contrast to the human gene where exon 2 contains a non-AUG-initiation site [15], [16], bovine exon 2 contains a regular start codon at this position. This bovine exon 2 is part of all transcripts identified here. However, an alternative start codon in exon 4 might be used when exon 3b is included into the transcript. Exon 3b is 4 nucleotides shorter than exon 3a and would cause a premature stop codon when translated from the upstream start codon. If the alternative start codon would be used, a truncated FNDC5 transcript like predicted for human FNDC5 would result (Figure 2B). Additionally, we detected a further 5′-variant which we named exon 3c. It spans exons 3a and b and includes additional 90 upstream nucleotides. We amplified four 3′-fragments which differed in usage of parts of exon 6 (Figure 2B). In total, our data revealed that at least 3 different 5′- and at least 4 different 3′-transcript variants exist in bovine skeletal muscle. Two full length transcripts could be amplified whereas attempts to amplify further transcripts in full length from muscle cDNA were not successful, probably due to the low abundance of these predicted transcripts. Our results however, demonstrated a higher number of FNDC5 transcript variants expressed in bovine skeletal muscle compared to humans and mice. No single nucleotide polymorphism was observed in FNDC5 cDNA in our sample.

**Figure 3:**
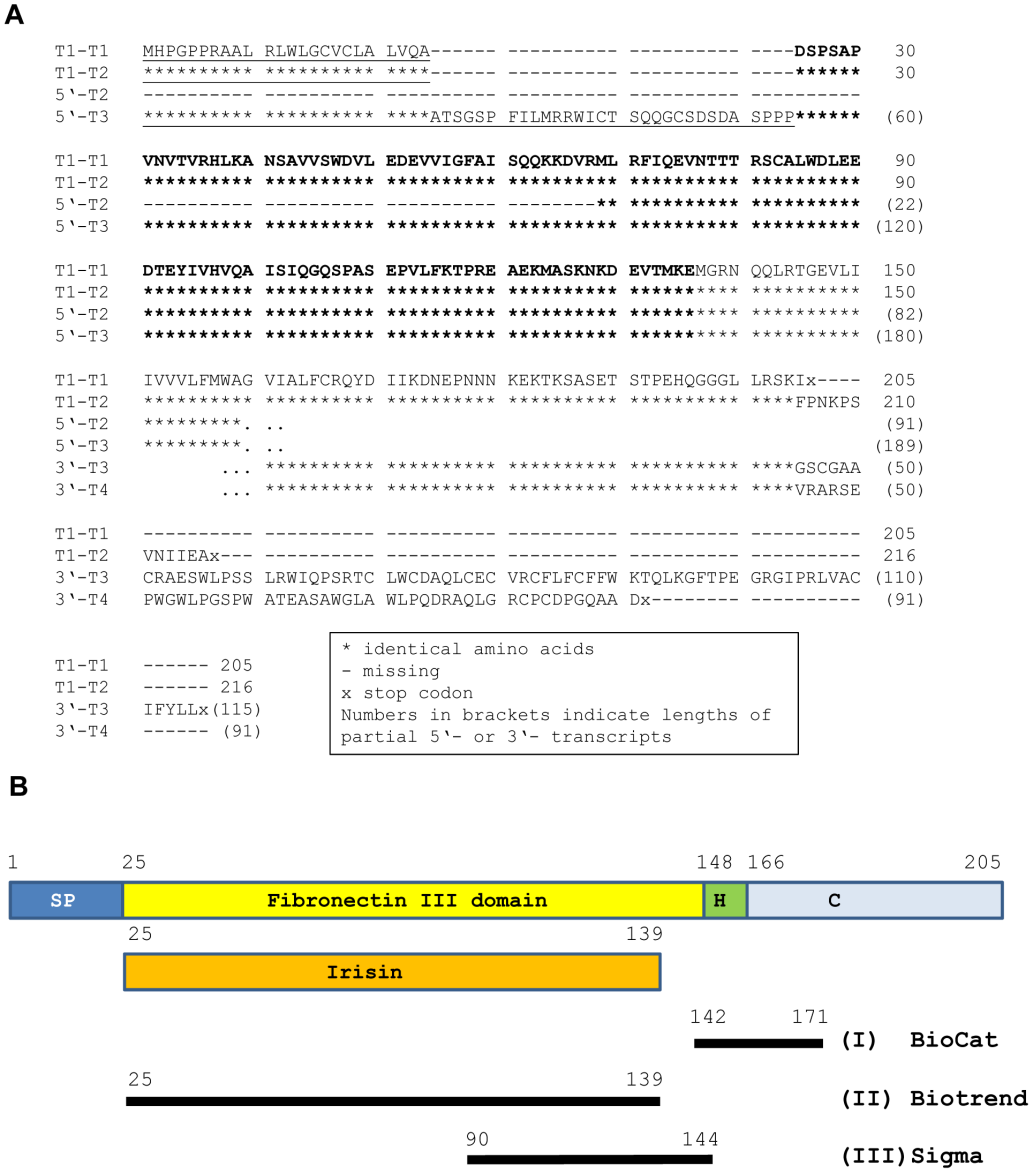
Bovine FNDC5 protein variants and antibody binding. (**A**) Deduced amino acid sequences for different transcripts and transcript fragments identified in this study. Putative signal peptides are underlined and the irisin peptide is marked in bold letters. Amino acids 25-204 in transcript T1–T1 are identical with murine and human amino acid sequences. (**B**) Schematic representation of antibody binding in the bovine FNDC5 protein and irisin peptide, respectively. The antibodies I, II, and III are further described in Materials and Methods. Annotation of functional domains and irisin was borrowed from the murine protein [1], [3]. SP: signal peptide; H: hydrophobic domain; C: C-terminal domain. Numbers refer to amino acids of bovine full length transcript T1–T1 (see A).

All identified 5′- and 3′-variants would affect the N- and C-terminal amino acid composition, respectively. The deduced amino acid sequences for the bovine transcripts and transcript fragments are given in Figure 3A. Beside two full length transcripts (T1–T1 and T1–T2), a further fragment (5′-T3) contained the complete sequence for irisin. In contrast, the fragment 5′-T2 would lack the first 44 amino acids of the protein and thus, would result in a truncated irisin protein. Translation of all transcripts provided, bovine FNDC5 variants could result in proteins with predicted molecular weights ranging from 16 to 34 kDa.

### FNDC5 mRNA is Expressed in Bovine Skeletal Muscle, Subcutaneous Adipose Tissue and Liver

FNDC5 mRNA abundance was determined in samples of LM from 20 bulls belonging to the groups high (n = 10) and low (n = 10) muscularity. Despite comparable live weight at slaughter, the groups differed significantly in protein and fat content of the carcasses (Table 1). Quantitative amplification of all putative bovine FNDC5 transcripts was ensured by selection of primers spanning parts of exons 3 and 4 which are present in all transcripts (Table 3). We observed a robust expression of total FNDC5 in muscle without differences between both groups (p>0.05).

Analysis of subcutaneous adipose tissue and liver from animals of both groups with the same primer pair revealed considerably lower expression of FNDC5 in both tissues compared to muscle. Again, no group differences were found (p>0.05).

We then analyzed the expression of the predicted transcripts separately. Transcripts with exon 3a (and putatively different 3′-ends; Figure 2B) did not reveal expression differences between the groups (p>0.05). All other transcripts (5′-T3, 3′-T3, 3′-T4; Figure 2B) could not be quantified due to expression close to the detection limit. However, all of them were present in all samples of both groups as revealed by gel electrophoresis of the RT-qPCR products (data not shown). These data confirmed that FNDC5 transcripts with different 5′- and 3′-regions were expressed in skeletal muscle of adult cattle at the same time.

### FNDC5 Protein is Expressed in Bovine Skeletal Muscle but does not Circulate in Plasma

**Figure 4:**
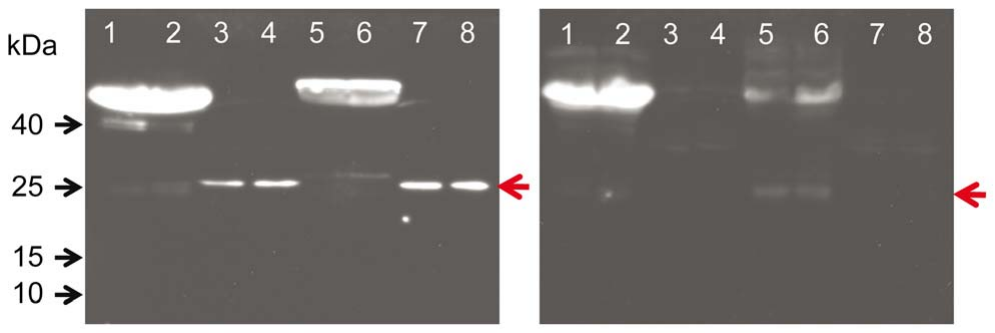
Characterization of the FNDC5 antibody. Western blot of mouse serum (1, 2), murine M. quadriceps femoris (3, 4), bovine plasma (5, 6), bovine M. longissimus (7), and bovine M. semitendinosus (8) with antibody against C-terminus of FNDC5 (left panel). The specific band of ∼25 kDa is marked by a red arrow. The right panel shows a parallel blot where the antibody was blocked with the recombinant antigen peptide prior to incubation. This antibody was raised against aa 149–178 of human FNDC5 corresponding to aa 143–171 in cattle (Figure 3, transcript T1–T1).

**Figure 5:**
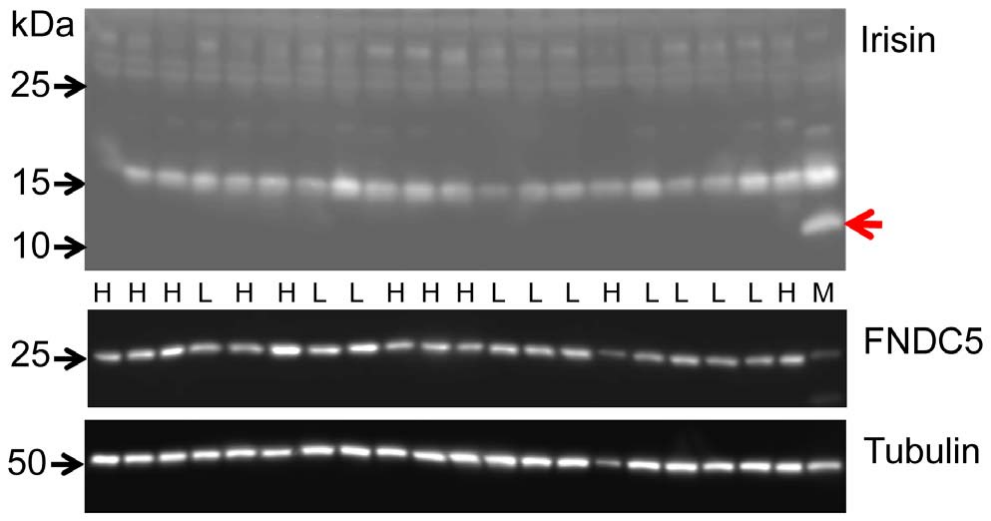
Detection of irisin and FNDC5 in bovine and murine skeletal muscle. Western blot of M. longissimus of bulls with high (H) or low (L) muscularity with an antibody against full-length irisin (upper panel). M: murine M. quadriceps femoris. The red arrow marks irisin at ∼12 kDa.The blot was stripped and re-incubated with an antibody against the C-terminus of FNDC5 (lower panel). A single, specific band was detected in bovine and murine muscle. Alpha-tubulin was used for normalization. No differences were found between the groups “low” and “high muscularity” (0.92±0.07 vs. 1.10±0.14; p = 0.27).

We detected FNDC5 protein in skeletal muscle of cattle with an antibody raised against the C-terminus (aa 149–178) of human FNDC5 (Figure 4, left panel). This antibody should detect full-length FNDC5 but not the peptide irisin (aa 32–143). A specific band at approximately 25 kDa was observed in skeletal muscle of cattle. A single band of the same size appeared in the murine muscle sample (M in lower panel of Figure 5), which served as a positive control. In cattle, the band was of similar strength in M. longissimus and in the superficial proportion of M. semitendinosus despite expected differences due to different contraction activity. The FNDC5 signal could completely be blocked by pre-soaking the antibody with the respective antigen peptide (Figure 4, right panel). In bovine plasma and murine serum however, no specific band was observed at this size (Figure 4). Analysis of LM samples from both cattle groups demonstrated a rather uniform FNDC5 protein abundance without quantitative differences between the groups (p>0.05; Figure 5, lower panel).

**Figure 6:**
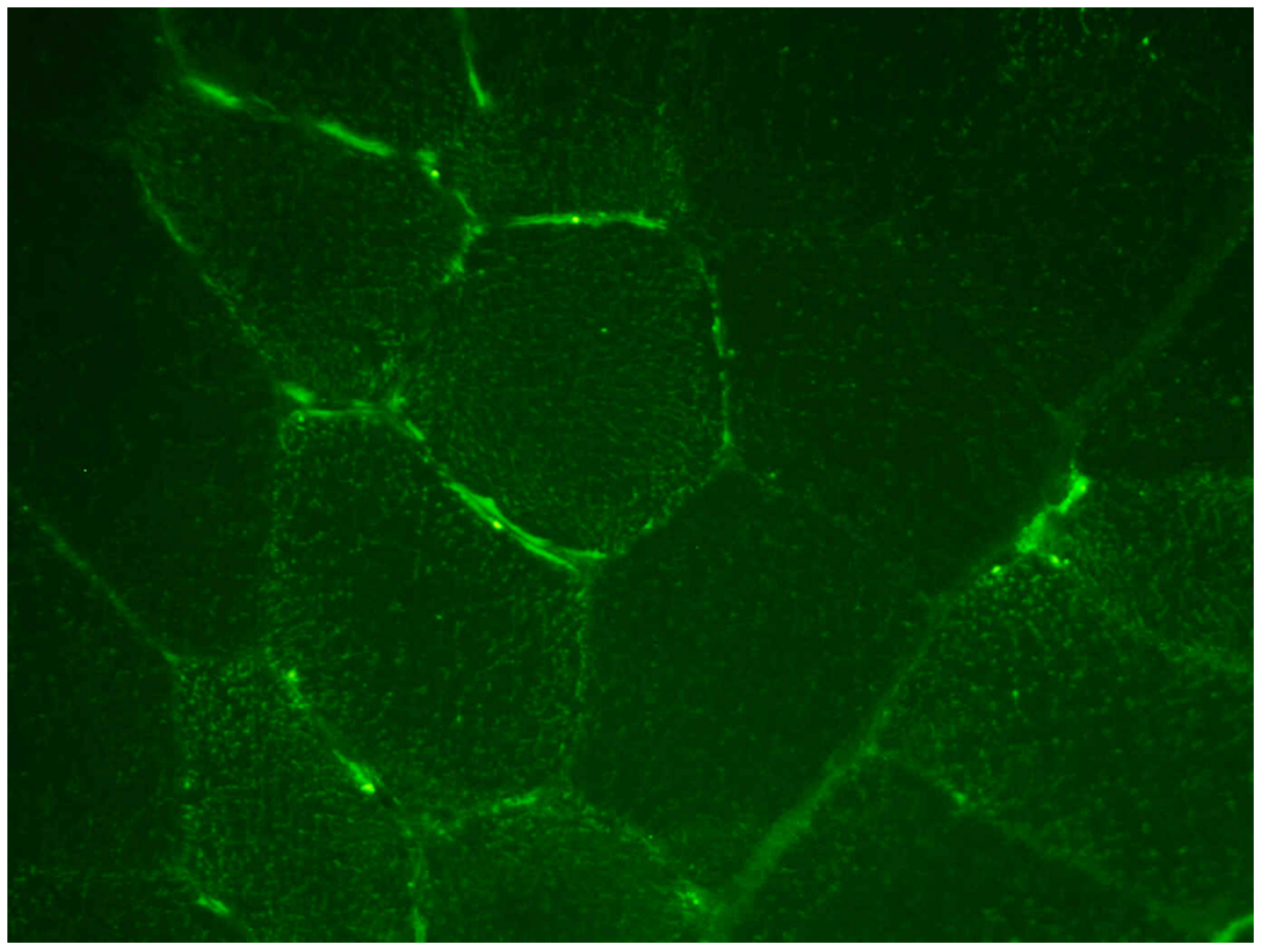
Cellular localization of bovine FNDC5 in skeletal muscle. Immuno-histochemical detection of FNDC5 in a cross-section of bovine M. semitendinosus (SM). FNDC5 was detected with the antibody against the C-terminus and was located at sarcolemma and in cytosol.

FNDC5 was localized in bovine skeletal muscle by immuno-staining in a cross-section of SM with the C-terminal antibody used before. We observed staining at the sarcolemma as expected for a transmembrane protein (Figure 6). Additional signals were present in the sarcoplasm as dots. Notably, not all muscle cells were positive for FNDC5. These data supported our observation of FNDC5 expression in adult, bovine skeletal muscle.

### Irisin Protein is not Detectable in Bovine Skeletal Muscle and Plasma

**Figure 7:**
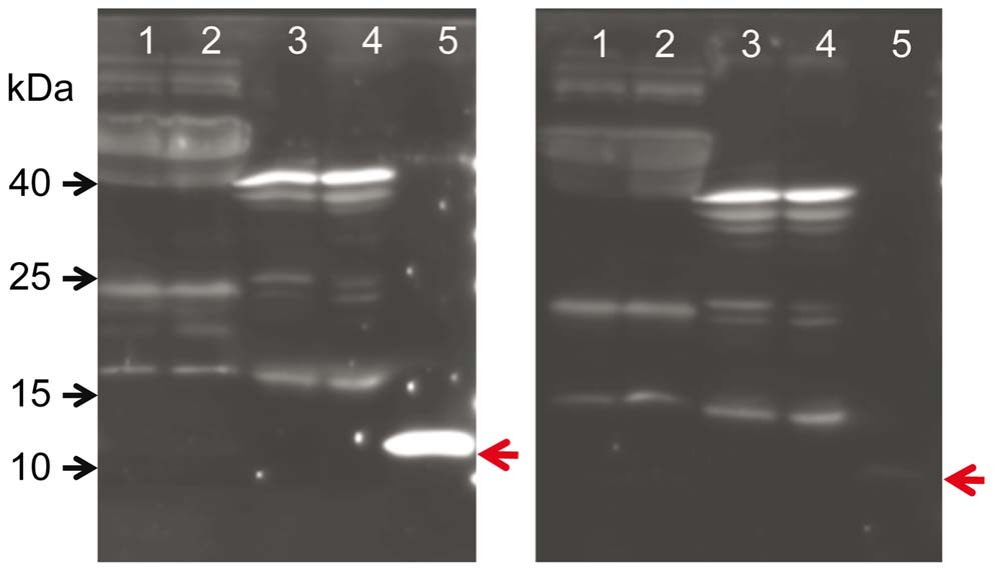
Characterization of the irisin antibody. Western blot of bovine plasma (1, 2), LM (3), and SM (4) with an antibody against full-length irisin. Recombinant irisin (5) was used as positive control (red arrow; left panel). The right panel shows a parallel blot where the antibody was blocked with recombinant irisin prior to incubation. The antibody was raised against aa 32–143 of human FNDC5 (corresponding to aa 25–136 in cattle; Figure 3, transcript T1–T1).

**Figure 8:**
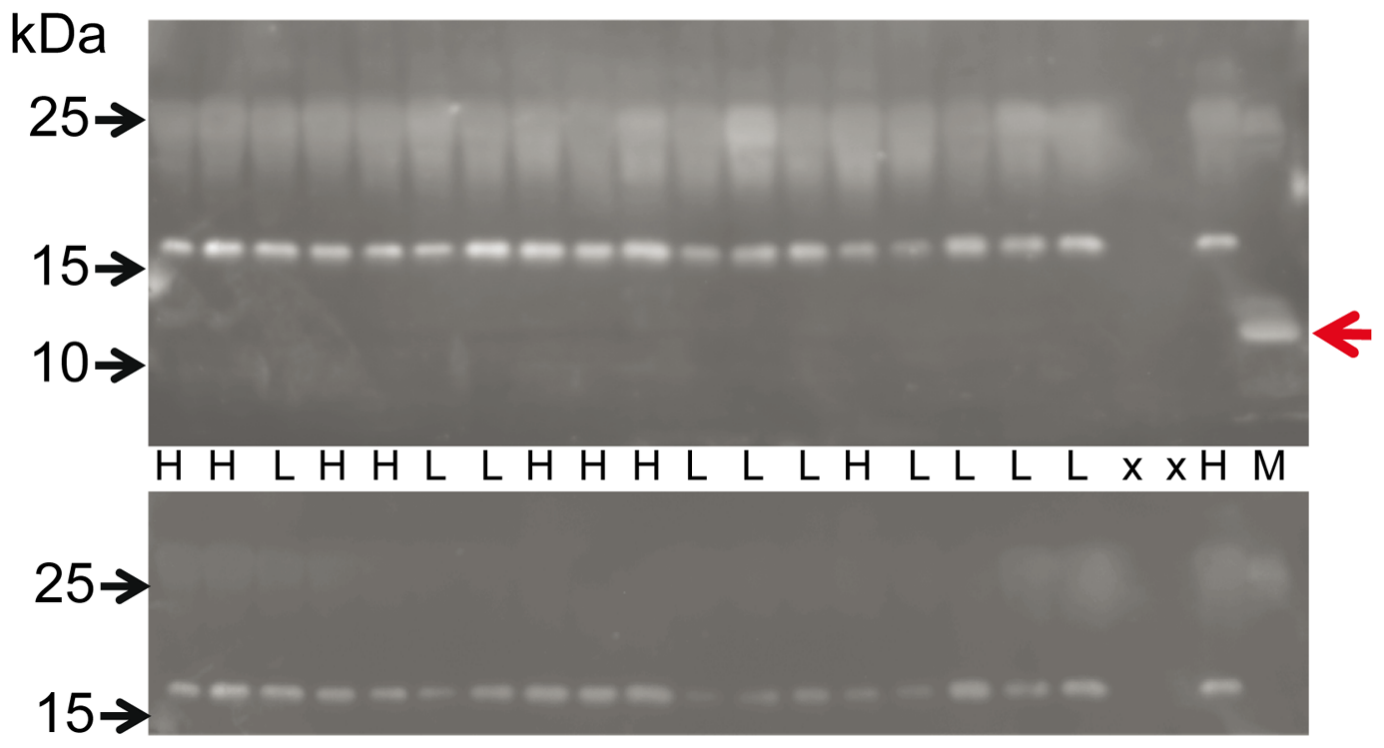
Detection of irisin in bovine plasma and murine serum. Western blot of plasma from bulls with high (H) or low (L) muscularity with an antibody against full-length irisin (upper panel). M: murine serum. x: empty lanes. A band specific for irisin was observed only in murine serum and is marked by a red arrow. The blot was stripped and re-incubated with the antibody against the C-terminus of FNDC5 (lower panel). A specific band was detected neither in bovine plasma nor in murine serum.

After removal of the signal peptide (aa 1–32), murine irisin is proteolytically cleaved from FNDC5, glycosylated and released into circulation [3]. Depending on the transcript, the bovine signal peptide is either 24 or 54 aa long (underlined in Figure 3A). The putative bovine irisin peptide comprises 112 aa with 100% identity to the peptide in mice and humans and has a calculated size of 12.6 kDa. In a first experiment, we did not detect a specific band neither at 12 kDa (irisin) nor at 25 kDa (FNDC5) in any sample with an antibody directed against a portion of irisin and the C-terminal FNDC5 sequence (Sigma-Aldrich, aa 90–144 see Figure 3B; data not shown). We used then a polyclonal irisin antibody which was raised against full-length recombinant irisin (Biotrend, aa 25–139, see Figure 3B). Western blot of bovine plasma and skeletal muscle however, did not reveal a signal at this size. Recombinant full-length irisin was used as positive control (Figure 7, left panel). In a parallel blot, where the antibody was pre-incubated with recombinant irisin, only this specific band was completely blocked (Figure 7, right panel). In bovine samples, no band was observed that could be blocked in the size range between 12 kDa and approximately 20 kDa. An irisin signal could be expected in this range considering larger size due to glycosylation of the peptide. The used antibody should also be capable of detection of FNDC5, because the putative irisin peptide is integral part of the full-length protein. Indeed we observed 2 weak bands between 20 and 25 kDa which were in the size range of the predicted proteins derived from transcripts T1–T1 (23 kDa) and T1–T2 (24 kDa, see Figures 2 and 3B). These bands disappeared when the antibody was blocked before (Figure 7, right panel). However, this observation was not reproducible in repeated analyses of our total sample. We used the same antibody in a western blot of bovine LM and plasma from animals with high (H) and low (L) muscularity (Figures 5 and 8, upper panels). There was no specific band for irisin observed in any cattle sample. To account for possible interference between removal of albumin in bovine plasma samples and subsequent irisin detection, purified and unpurified plasma samples were compared at different concentrations by western blot. Irisin was not detectable under any condition (data not shown). In contrast, the positive control (murine M. quadriceps femoris [M]) revealed a distinct band at 12 kDa, which was specific for irisin (red arrow in upper panel of Figure 5). Our results demonstrated that the hormonal active peptide irisin is present in muscle of sedentary mice but not in bovine skeletal muscle. Furthermore, the co-existence of full-length FNDC5 and its cleaved peptide irisin was shown here in the murine control (Figure 5), and irisin was also detected in serum of this mouse (red arrow in upper panel of Figure 8). In contrast, irisin was not detectable in plasma of our investigated F_2_-bulls (Figure 8).

## Discussion

Recently, FNDC5 - a hitherto largely disregarded transmembrane protein - was identified as source of a messenger initiating a “browning” program in subcutaneous adipose tissue [3]. This study put FNDC5 in the spotlight of research related to obesity and diabetes. Recent results showed that circulating irisin levels correlated with muscle mass and body mass index in humans [5], [11], [12] and we raised the question whether similar relations exist in cattle. We provided here a gene model for the bovine FNDC5 locus by correcting and expanding the automatic annotation of this gene in the current genome build. On this basis, we analyzed FNDC5 at mRNA and protein level in two groups of cattle exhibiting strongly different muscularity and adiposity traits.

### The Bovine FNDC5 Locus is Similarly Organized Like in Humans and Mice but more Transcript Variants are Expressed in Bovine Muscle

We resolved the striking difference between FNDC5 annotation in the current bovine genome build (UMD 3.1) and that in the human genome (Figures 1A, B). The general structure of bovine FNDC5 resembles those of the human and murine genes. The similarity of the coding regions at DNA level is greater than 90% between the three species leading to 100% identity in a string of more than 180 amino acids. Consequently, all but one putative bovine transcripts encode 112 amino acids for the peptide irisin that was described as fully conserved over a wide range of species [3]. The human gene however, lacks a regular start codon and is likely to use a non-AUG-initiated N-terminal extension. This feature is unique to humans. Even chimpanzee and gorilla, like any other mammal, possess a regular AUG-signal at this position indicating that the AUA-signal evolved rather recently [15]. The genomic basis for the N-terminal amino acid sequence of human FNDC5 given by Boström et al. [3] remains unclear, however [16].

The bovine genome assembly UMD 3.1 contains a gap within the FNDC5 locus (Figure 2A). A gap is present at almost the same position in the porcine FNDC5 locus on chromosome 6 in the current genome build (Sscrofa 10.2). We speculate that repetitive elements may be present at this position in the genomes of both ungulate species.

In our study, a robust expression of total FNDC5 mRNA was demonstrated in M. longissimus of adult cattle. Beside 2 different full-length transcripts, further 5′- and 3′variants were detected in skeletal muscle and indicate a higher transcript variability than in humans and in mice. Data from the EST collections indicated more 3′-transcript variants in the human gene than currently annotated. In mice however, no data support the existence of transcript variants so far.

The effect of exercise on mRNA abundance of FNDC5 was questioned by Timmons [13]. In reply, Boström et al. [26] accentuated that induction of PPARGC1A is prerequisite for increased FNDC5 mRNA expression. A significant increase of PPARGC1A and FNDC5 mRNA abundance was noticed in cultured human rhabdomyosarcoma cells after treatment with a combination of omega-3 fatty acids for 24 or 48 hours [27]. This study indicated that PPARGC1A/FNDC5 induction does not exclusively depend on physical exercise. Investigations in humans and pigs did not reveal a systematic effect of exercise on FNDC5 expression and thus support this notion [14], [28]. Instead, increased FNDC5 mRNA abundance was noticed in obese humans and rats [5], [29]. However, an association between body composition and FNDC5 expression was not observed in cattle of our study. Microarray data from skeletal muscle of the bulls analyzed in our study did not reveal a significant expression of PPARGC1A (data not shown). This may explain the uniform FNDC5 mRNA abundance observed in both groups of cattle.

Notably, we have qualitatively demonstrated the mRNA expression of two different transcripts in skeletal muscle, which use different 5′-exons with alternative start codons. Furthermore, our data provide evidence for the simultaneous transcription of variants with different cytoplasmic domains of the protein (see Figures 2 and 3). It remains to be elucidated, whether all transcripts are translated, and if so, which functions the resulting FNDC5 protein variants would have. FNDC5 is weakly expressed in bovine subcutaneous adipose tissue and liver. This is in line with results in humans, mice and rats [3], [17], [30]. Roca-Rivada et al. [17] found FNDC5 protein in the secretome of muscle and fat cells. For this reason FNDC5 enlarges the list of proteins which are termed adipo-myokines and may mediate the cross-talk between skeletal muscle and adipose tissue [17], [18], [31].

### FNDC5 is Abundant in Bovine Skeletal Muscle but Neither FNDC5 nor Irisin Circulate in Bovine Plasma

We demonstrated FNDC5 protein expression in bovine skeletal muscle as a single band with an antibody specifically detecting full-length FNDC5 but excluding irisin. However, several peptides in the size range from ∼16 up to 30 kDa were expected since the antibody had the capability of detecting all resulting protein variants. Our results thus indicate that one or two transcripts with similar size were translated preferentially. Abundance of FNDC5 protein was not related to variation in body fat content and muscle mass in cattle under our experimental conditions.

Boström et al. [3] detected a protein of ∼22 kDa in murine serum and human plasma and named it irisin. Subsequent studies in humans, mice and rats demonstrated effects of energy expenditure characteristics in humans [32], fasting/re-feeding regimens, obesity and exercise in rats [17] and chronic kidney disease in humans [21] on circulating proteins of this size range (western blot; 20–26 kDa), whereas no effects of calorie restriction in rats on FNDC5 protein levels in plasma were found [20]. In contrast, we did not detect a specific band for FNDC5 in bovine plasma. This indicates that full-length FNDC5 was bound to cells as expected for a transmembrane protein and was not secreted into circulation.

It should be noted that irisin secretion is assumed to result from proteolytic cleavage of membrane-bound FNDC5 [3]. This means that irisin secretion might not necessarily be preceded by up-regulation of FNDC5 mRNA or protein. However, we were not able to detect irisin at its expected size (∼12 kDa) in muscle or plasma from animals of neither experimental group. However, a specific band for irisin was found in muscle and serum of a mouse used as control. This is of particular interest since Roca-Rivada et al. [17] noted, that all studies on irisin, including those of Boström et al. [3], failed to show a peptide corresponding to the expected size of irisin (12.6 kDa). The band shown as positive control in this study could completely be blocked by pre-incubation with recombinant irisin in a larger sample of mice thus proving its specificity (Brenmoehl, unpublished data). Interestingly, irisin was present in murine skeletal muscle, where it should be cleaved from full-length FNDC5 and released into circulation after glycosylation [3]. Our results therefore support this proposed mechanism in mice.

Collectively, our findings demonstrated that FNDC5 resides in bovine skeletal muscle but neither FNDC5 nor irisin circulated in bulls of the Charolais × Holstein F_2_-population under standard husbandry conditions despite large variation in body composition.

### Irisin and FNDC5 Protein in Muscle and Circulation – Many Open Questions

Numerous studies tried to elucidate the potential role of FNDC5/irisin in metabolism and disease in different species in the past 18 months. Beside some answers given - also with our results - several basic questions were left unanswered or were even raised with new results. Some of them will be addressed below.

#### Is full-length FNDC5 released into circulation?

Our negative results contradict observations of basal FNDC5 levels circulating in rodents and humans [3], [4], [20], [21], [26], [33]. These results leave the question to be answered, how a classical transmembrane protein can be released as entity from skeletal muscle. Huh et al. [5] suggested that exercise-induced muscle damage might be responsible for acute FNDC5 release. Further mechanisms to explain circulating FNDC5 still await discovery.

#### Does irisin exist?

The positive control for murine irisin – backed by further data [Brenmoehl, unpublished data] – provide first evidence for circulating irisin at its expected size (12.6 kDa). Our results in cattle however, are in line with other studies failing to show irisin [3], [4], [17], [20], [21]. Furthermore, the existence of full length FNDC5 transcripts and thus, the basis for irisin cleavage in humans was questioned and FNDC5 was suggested as transcribed pseudogene [16]. However, FNDC5 protein (∼23 kDa) was demonstrated in human muscle and adipose by western blot [3], [17] and important functions of FNDC5 in neuronal development are likely [2], [34], [35]. Recent structural analyses of recombinant irisin indicated that it forms a continuous intersubunit ß-sheet dimer [36] and could thus explain the detection of 25 kDa bands when appropriate antibodies had been used.

#### What do commercial ELISA kits for irisin detect?

Our data from western blots with one of the antibodies used in the kits demonstrated a high degree of unspecific binding in bovine plasma and murine serum besides specific detection of irisin. These kits were used in numerous human studies with partly highly different measured levels [5], [6], [11], [12], [21], [30], [37]–[44]. It cannot be excluded that this variation was influenced by unspecific binding of the antibodies. Consequently, a validation of all antibodies used in ELISA kits by quantitative western blot analysis was demanded by Erickson [45]. The results of Raschke et al. [16] underlined this urgent demand.

### Conclusion

Our results provide first data on FNDC5 expression in cattle and a valid model for this hitherto uncharacterized bovine gene. The uniform mRNA expression as well as the abundance and distribution of FNDC5 protein in skeletal muscle of bulls with largely differing body composition indicate no relationships between FNDC5, muscularity and adiposity traits in our experimental cattle. Neither FNDC5 nor its putatively secreted peptide irisin were found in circulation and indicate functional differences of FNDC5 regulation between rodents and cattle. Evidence for irisin as 12 kDa peptide in murine skeletal muscle and serum may contribute to a process of re-evaluation of recent studies on FNDC5 and irisin.
